# Bridging Clinical Outcomes and Cost‐Effectiveness: The Role of Real‐World Data in Cystic Fibrosis Therapy

**DOI:** 10.1002/prp2.70138

**Published:** 2025-06-17

**Authors:** Ida Štimac, Andrea Katrin Faour, Elvira Meni Maria Gkrinia, Andrej Belančić

**Affiliations:** ^1^ Faculty of Medicine, University of Rijeka Rijeka Croatia; ^2^ Independent Researcher Vancouver British Columbia Canada; ^3^ Independent Researcher Athens Greece; ^4^ Department of Basic and Clinical Pharmacology and Toxicology Faculty of Medicine, University of Rijeka Rijeka Croatia

**Keywords:** cystic fibrosis, orphan medicine, pharmacoeconomics, rare disease, real‐word evidence

**Abbreviations**
BMI: body mass index
CEA: cost‐effectiveness analysis
CF: cystic fibrosis
CUA: cost‐utility analysis
DMD: disease‐modifying drug
ETI: elexacaftor/tezacaftor/ivacaftor
FEV_1_
: lung function
HTA: health technology assessment
PROs: patient‐reported outcomes
QoL: quality‐of‐life
RCT: randomized controlled trial
RW: real‐world
SLR: systematic literature reviews
SMA: spinal muscular atrophy
UHC: universal health coverage

We would like to commend the authors, Perrotta et al., for their insightful contribution published in *Pharmacology Research and Perspectives* on the real‐world (RW) outcomes associated with elexacaftor/tezacaftor/ivacaftor (ETI) in patients with cystic fibrosis (CF). This retrospective observational study analyzed clinical data from 139 patients with CF treated with ETI at the CF Reference Centre at the Umberto I Hospital at Sapienza University of Rome. Patients were 12 years of age or older and had a confirmed diagnosis of CF, either with homozygous or heterozygous F508del mutations in the CFTR gene [1]. The authors reported statistically and clinically significant improvements across all primary outcomes, with mean increases in lung function (FEV_1_) and body mass index (BMI), and a notable reduction in pulmonary exacerbation rates [1]. These improvements were observed regardless of prior CFTR modulator use, underscoring the broad therapeutic effectiveness of ETI in a RW setting [1].

Despite the promising results, the study design bears limitations. Its retrospective, single‐center design limits causal inference and is prone to information and selection biases. The absence of a control group restricts comparative analysis, while the lack of subgroup analyses such as by disease severity or comorbidities limits insight into differential responses. Furthermore, only half of the included patients completed 2 years of follow‐up after treatment initiation, thereby limiting the ability to capture long‐term benefits and adherence. To address these limitations, prospective, multicenter studies with longer follow‐up are warranted. Additionally, incorporating quality‐of‐life (QoL) metrics and standardized adverse event reporting would strengthen the evidence on ETI in clinical practice. These considerations highlight the need for expanded RW evidence and post‐marketing surveillance would also further inform health economic assessments [1].

The purpose of this commentary is to draw further attention to this gap in the literature, propose the potential design of future studies, and explore potential additional benefits of ETI, particularly from a cost‐effectiveness and pharmacoeconomic perspective. We also aim to do this by drawing parallels with the extensive investigations into outcomes and economics in the spinal muscular atrophy (SMA) field, as presented widely by Belančić et al. across a broad spectrum of recent publications, and by sharing the knowledge gained in the rare disease and SMA fields to inform and support its implementation in the context of CF.

Importantly, randomized controlled trials (RCTs) and RW evidence can be considered complementary, akin to yin and yang, a concept especially relevant in the context of rare diseases. While RCTs offer internal validity by studying small, homogeneous populations under controlled conditions, RW evidence reflects the more heterogeneous, day‐to‐day clinical environment and can capture long‐term, practical outcomes that RCTs often miss. RW evidence is essential not only for assessing treatment effectiveness but also for long‐term safety monitoring. It complements spontaneous adverse drug reaction reporting systems like EudraVigilance and the FDA Adverse Event Reporting System, enabling trend analyses and subgroup‐specific safety evaluations. As highlighted in recent literature, there is growing advocacy for healthcare systems to act as “bodyguards of drug safety,” proactively identifying and managing post‐marketing risks [2].

Systematic literature reviews (SLRs) combined with pharmacoeconomic modeling and newly developed disease‐modifying drugs (DMD) switch models can identify substantial cost savings in the setting of CF by incorporating longer term prospective outcomes. Reassessment of orphan drugs and their indications may also inform updates to national health insurance reimbursement guidelines, thereby resulting in significant reductions in healthcare expenditures [3, 4]. Belančić et al. [5] estimated that Europe could save up to €905.5 million over 5 years by switching newly diagnosed patients with SMA type 1 from nusinersen to risdiplam. This switch is supported by risdiplam's clinical and practical advantages, including oral administration, improved safety and tolerability, and greater acceptance by patients and caregivers, all of which may enhance treatment satisfaction [5]. Similarly, in CF, patients are increasingly switching from older CFTR modulators (e.g., ivacaftor, lumacaftor/ivacaftor) to the more effective ETI, offering an opportunity to evaluate RW cost‐effectiveness and treatment optimization. Generally, orphan medicines are compared to best supportive care, with few direct head‐to‐head studies available. This limits evidence‐based policymaking and underscores the need for RW data to inform decisions on disease control, adverse effects, and QoL in rare diseases [5]. Additionally, the value of RW evidence extends beyond patients who switch therapies. It is equally crucial for those who remain on their initial DMD. For example, the cost savings observed in the switch from nusinersen to risdiplam in SMA type 1 highlight how RW data can reveal opportunities for economic efficiency and improved adherence. However, such insights could also apply to non‐switch cohorts, offering guidance for optimizing long‐term treatment strategies [5].

Unlike clinical trials, which require controlled environments, strict inclusion/exclusion criteria, and extensive monitoring, RW studies offer the possibility of rapid access to data with fewer resources and at a lower cost, reflecting broader and more diverse patient populations [6]. Although RW data has not traditionally played a major role in regulatory processes, recent developments signal growing acceptance by national authorities [7, 8]. Most cost‐effectiveness analyses (CEA) are based on data extrapolated from clinical trials, which may not fully reflect RW healthcare settings. Therefore, RW evidence can bridge this gap. The data from Perrotta et al. can be used to validate or adjust existing cost‐effectiveness models, especially those that lack long‐term RW outcome data. In addition to its significance for regulatory matters such as pricing, reimbursement, and allocation of financial resources, RW evidence also enhances clinical care for patients with CF by providing practical experience. This ultimately enhances patients' QoL and informs management decisions, particularly when initial DMD prove ineffective or poorly tolerated [9, 10].

Clinical efficacy underpins the economic value of ETI, which has demonstrated significant improvements in FEV_1_, BMI, and reductions in pulmonary exacerbations, even among patients with advanced CF [11, 12, 13]. These clinical benefits support assumptions of quality‐adjusted life year gains and decreased healthcare utilization, both of which are key components of model‐based economic evaluations. In a SLR of pharmacoeconomic evaluations for orphan medicines used to treat SMA, Belančić et al. found that these medicines were not cost‐effective in multiple studies and across various settings [14]. Although orphan medicines may deliver substantial health gains, they are rarely found to be cost‐effective. This raises an important question: are conventional measures, such as willingness‐to‐pay thresholds, truly appropriate for evaluating treatments for rare diseases, including CF? The findings of Perrotta et al. confirm the high therapeutic value of ETI and this in turn highlights the necessity of revising cost‐effectiveness thresholds for orphan medicines to enhance their accessibility and integration into routine clinical practice [1, 14].

CEAs and cost‐utility analyses (CUAs) are critical in evaluating orphan medicines, even when treatment costs are high and incremental cost‐effectiveness ratios exceed conventional thresholds applied to standard therapies [14, 15, 16]. Within health technology assessment (HTA), pharmacoeconomic evaluation represents only one dimension. Ethical, social, and equity considerations are equally vital, particularly for orphan medicines. Therefore, policymakers must integrate these broader factors when developing treatment strategies. Incorporating QoL measures and patient‐reported outcomes (PROs) is essential in assessing therapies for rare diseases, despite the inherent challenges [14, 15, 16]. Although CEAs and CUAs facilitate value comparisons among treatments for the same indication, the interpretation of these data should also consider emerging clinical evidence and evolving knowledge to guide optimal, patient‐centered decision‐making [14, 15, 16].

Healthcare systems differ widely across countries, affecting the generalizability of RW evidence and policy decisions. Most European countries, including Italy, operate under universal health coverage (UHC) models that ensure near‐complete access to healthcare services, with private insurance playing a supplementary role [17]. In contrast, the United States primarily relies on a private, market‐based insurance system, resulting in millions of uninsured individuals. Consequently, insights from the Italian healthcare setting may inform policy and reimbursement decisions in countries with similar UHC frameworks but have limited applicability to the US private healthcare context [18]. The United Nations recognizes UHC as a critical global priority, emphasizing the need to address scientific, technical, and managerial challenges to achieve equitable access worldwide [19]. Despite the demonstrated clinical and economic benefits of ETI in CF, global access remains limited. Presenting robust RW outcome data can support more equitable access, advocate for broader inclusion of ETI in national formularies, and promote more affordable pricing strategies [20].

In conclusion, the RW evidence presented by Perrotta et al. reinforces the substantial clinical benefits of ETI for patients with CF, including those with advanced disease. While the retrospective, single‐center design and limited follow‐up highlight the need for more robust, multicenter, and prospective studies, these findings underscore the importance of integrating RW data into HTAs and economic evaluations. Such evidence not only validates clinical trial outcomes but also informs cost‐effectiveness models, reimbursement policies, and broader access initiatives, particularly in the context of orphan medicines where traditional thresholds may be inadequate. Policymakers should consider ethical, social, and equity dimensions alongside economic analyses to optimize treatment strategies for rare diseases. Furthermore, global disparities in ETI access persist, emphasizing the need for ongoing RW data collection to support equitable inclusion in national formularies and to advocate for more affordable pricing. Ultimately, a comprehensive approach that incorporates clinical outcomes, PROs, and health system variability will be essential to maximize the impact of innovative therapies such as ETI in routine clinical practice worldwide.

## Author Contributions

**Ida Štimac:** writing – original draft, writing – review and editing, investigation. **Andrea Katrin Faour:** writing – review and editing, writing – original draft, investigation. **Elvira Meni Maria Gkrinia:** writing – review and editing, writing – original draft, investigation. **Andrej Belančić:** conceptualization, investigation, writing – original draft, writing – review and editing, project administration, supervision.

## Ethics Statement

The authors have nothing to report.

## Conflicts of Interest

The authors declare no conflicts of interest.

## Data Availability

Available upon reasonable request sent to the corresponding author.
